# Application of mevalonolactone prevents deterioration of epidermal barrier function by accelerating the lamellar granule lipid transport system

**DOI:** 10.1111/srt.13202

**Published:** 2022-09-23

**Authors:** Bin Chen, Nan Lu, KeeSuh Lee, Lei Ye, Chiho Hasegawa, Kazuhisa Maeda

**Affiliations:** ^1^ Pechoin Biotech Co. Ltd. Shanghai China; ^2^ Tokyo University of Technology Hachioji Japan

**Keywords:** ABCA12, free fatty acid, improving dry skin, lamellar granule, mevalonolactone, PPARbeta/delta

## Abstract

**Background:**

Fatty acids increase ATP‐binding cassette ABC transporter A12 (ABCA12) levels via an increase in peroxisome proliferator‐activated receptor β/δ (PPAR β/δ). Promoting lipid transport to lamellar granules has been suggested to improve epidermal barrier function in patients with dry skin.

**Objective:**

We investigated whether mevalonolactone (MVL) produced by *Saccharomycopsis fibuligera* improves dry skin by promoting ABCA12 expression and the amount of free fatty acids in epidermal keratinocytes.

**Methods:**

We examined whether MVL increases ABCA12 mRNA and protein levels and the amount of Nile red‐positive lipids in cultured epidermal keratinocytes and in a three‐dimensional epidermal model by cell staining. Promotion of fatty acid production by MVL was analyzed by liquid chromatography‐mass spectrometry. We also evaluated whether MVL addition increases PPAR β/δ mRNA expression in cultured keratinocytes. Based on the results, a randomized controlled trial was conducted in which milky lotions containing MVL and placebo were applied to dry facial skin of healthy female volunteers in winter.

**Results:**

MVL increased ABCA12 mRNA and protein levels and lamellar granule number and size. Fatty acid analysis revealed that MVL elevated myristic acid, palmitic acid, and palmitoleic acid levels as well as PPAR β/δ mRNA expression. In human tests, milky lotions containing MVL were shown to significantly improve transepidermal water loss (TEWL) in the stratum corneum compared to placebo.

**Conclusion:**

The results suggest that MVL increases fatty acid uptake and ABCA12, promotes fatty acid transport to lamellar granules, and improves epidermal barrier function in dry skin through increased expression of PPAR β/δ.

## 1 INTRODUCTION

The epidermis, which is the most superficial layer of the skin, plays an important role in protecting the body from external stimuli and dehydration. Intercellular lipids of the horny layer, including ceramide (37%), cholesterol (32%), long‐chain fatty acids (16%), and cholesterol esters (15%), have critical functions in maintaining the barrier function of the skin.^1^, ^2^, ^3^ When the skin's ability to generate these molecules is decreased, the barrier function is reduced, rendering the skin more susceptible to the effects of external stimuli and dehydration. This state can readily lead to skin problems, such as dry or sensitive skin.^4^ High skin sensitivity may result in pain, tingling, skin irritation, and other sensitive skin‐related conditions, including acne, rash, and atopic dermatitis. Skin care products, which are primarily aimed at improving moisture retention and the barrier function of the stratum corneum, are used as treatments for sensitive skin. Intercellular lipids of the stratum corneum are largely responsible for moisture retention and the stratum corneum's barrier function.^5^ Intercellular keratinocyte lipids are produced from precursor lipids, which are stored in the lamellar body, an intracellular organelle of the stratum granulosum.^6^ The number of lamellar granules increases with differentiation of epidermal keratinocytes, and these granules occupy one‐third of the cytoplasm of the stratum granulosum.^7^ When the stratum granulosum differentiates into the stratum corneum, the lamellar granule membrane fuses with the stratum granulosum membrane at the boundary between the stratum granulosum and the stratum corneum, releasing lipids in lamellar granules.^8^ Synthesis of lamellar bodies is regulated by the surrounding calcium gradient in the stratum granulosum of the upper epidermis.^9^ When the barrier is disrupted, the amount of calcium surrounding the cells in the stratum granulosum decreases, and this change in calcium concentration appears to be the primary signal that induces lamellar body secretion and regeneration.^9^ Ceramide is synthesized through acid amide bond formation between fatty acids and sphingoids. Ceramide in the skin is characterized by greater than 50% long‐chain fatty acid binding^10^, ^11^ and is released from lamellar granules, and cholesterol is derived from the cell membranes of granular cells, but the mechanism of free fatty acid secretion remains unclear.

Free fatty acids promote differentiation of epidermal keratinocytes, playing an important role in proper formation of the horny layer.^12^ Free fatty acids thus help to maintain healthy skin and mitigate skin damage.^13^ Unsaturated fatty acids are particularly necessary for maintaining the moisture content, flexibility, and smoothness of the stratum corneum and protecting the skin from damage.^13^ Decreased levels of free fatty acids can cause improper metabolism in the epidermis, which can in turn lead to problems such as scaling, internal hemorrhage from fragile capillaries, reduced wound healing, and dry skin due to increased moisture loss.^14^, ^15^, ^16^ Dry skin is particularly common in winter months, especially among elderly individuals, as the effects of water loss from the stratum corneum are compounded by age‐related thinning of the skin. Exacerbation of dry skin can lead to conditions such as xeroderma and atopic dermatitis. Moreover, atopic dermatitis, eczema, dry skin, and xeroderma are associated with increased transepidermal water loss (TEWL) and are related to decreased levels of intracellular lipids in the horny layer, including decreased ceramide and unsaturated fatty acid levels.^17^, ^18^ The fatty acid composition of keratinocytes is altered in inflammatory skin diseases such as atopic dermatitis and psoriasis, and it has been speculated that fatty acids are involved in regulating inflammation.^19^, ^20^, ^21^, ^22^, ^23^, ^24^

ATP‐binding cassette subfamily A member 12 (ABCA12) is a critical transmembrane lipid transfer protein in keratinocytes that plays an important role in lamellar granule lipid transport.^25^ ABCA12 is an example of an ATP‐binding cassette transporter that uses energy liberated from ATP hydrolysis to transport various molecules across the plasma membrane or vesicular walls.^25^ Spontaneous ABCA12 mutation has been shown to interfere with lipid transport into lamellar granules, thus causing ichthyosis.^25^ ABCA12 is expressed between the stratum spinosum and stratum granulosum, and this transporter helps to localize and store synthesized intracellular lipids within lamellar granules and eventually secrete stored lipids from cells into the intercellular space of the stratum corneum.^26^ ABCA12 expression is reported to increase in response to PPAR α and PPAR β/δ.^27^ Nile red stains unsaturated fatty acids, cholesterol, cholesteryl esters, some triglycerides, and phospholipids^28^, ^29^ as well as intracellular lipid droplets.^30^ In keratinocytes, the granules stained by Nile red are lamellar bodies.^31^

**FIGURE 1:**
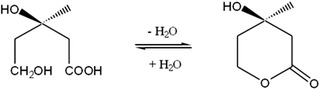
Chemical structures of mevalonic acid (MVA) and mevalonolactone (MVL)

The purpose of this study was to examine substances that may promote generation of free fatty acids in epidermal keratinocytes. We cultured a 3D human epidermis model with mevalonolactone (MVL; Figure 1) and measured TEWL using a Tewameter to assess effects on barrier function in this 3D human epidermis model of the human epidermis. We found that MVL promotes uptake of free fatty acids and improves barrier function. MVL is a product of mevalonic acid (MVA) dehydration, and it reverts to MVA when in contact with water. MVA is a precursor of various steroids and isoprenoids, including vitamin K, CoQ10 (ubiquinone), cholesterol, and squalene. Moreover, application of MVA to aging mouse skin was shown to restore lost cholesterol synthesis capacity.^32^ Disruption of barrier function by topical acetone treatment results in an increase in synthesis of free fatty acids, sphingolipids and cholesterol in living layers of the epidermis, leading to barrier repair.^33^ Acute barrier disruption causes an increase in the rate‐limiting enzymes β‐Hydroxy β‐methylglutaryl‐CoA (HMG CoA) reductase and serine palmitoyl transferase.^33^ Repeated application of lovastatin, which suppresses the action of HMG‐CoA reductase, inhibits conversion of HMG‐CoA to MVA in untreated skin, resulting in disturbed barrier function accompanied by increased DNA synthesis and epidermal hyperplasia.^33^ Cholesterol replacement and topical treatment with lovastatin, which inhibits accumulation of metabolites of the MVA pathway, have the potential to alleviate porokeratosis.^34^ In this study, we investigated the effects of MVL on stratum corneum formation and its mechanism of action using cultured keratinocytes and a 3D human epidermis model. We also investigated the efficacy of MVL‐containing cosmetics when used continuously in humans.

## 2 MATERIALS AND METHODS

### 2.1 Materials

In this study, we used a natural form of MVL (ADEKA LUMINACARE Mevalonolactone; ADEKA Corporation, Tokyo, Japan) purified from the culture supernatant of *Saccharomycopsis fibuligera*.

### 2.2 Effects of MVL on the amount of immunoreactive ABCA12 protein and Nile red‐positive lipid content in human keratinocytes

Chamber slides were seeded with human keratinocytes (Cell Lines Service, Eppelheim, Germany; 50,000 cells/500 µl), and the cells were cultured for 3 days in the presence or absence of 0.05% (3.84 mmol/L) MVL. The samples were washed with phosphate‐buffered saline (PBS). A 4% paraformaldehyde‐PBS solution was added, and the samples were incubated for 20 min before being washed with PBS. Then, 10% normal goat serum was slowly added, and the solution was allowed to stand for 30 min before washing with PBS. Next, 1 µg/ml of anti‐ABCA12 polyclonal antibody (Alexa Fluor 488 conjugated, Bioss Inc., Boston, MA) was added, and the solution was allowed to stand for 90 min before washing with PBS. The nuclear stain 4',6‐diamidino‐2‐phenylindole (DAPI, Thermo Fisher Scientific Inc., Waltham, MA) was added, and the sample was allowed to stand for 5 min before washing with PBS, followed by washing with pure water. Mounting agent was added, and a glass coverslip was placed on the sample. The sample was observed by fluorescence microscopy with a WIB longpass fluorescence cube (excitation wavelength: 460–495 nm [blue], detectable fluorescence wavelength: 510 nm or more [green to red]). Nile red solution (500 µg/ml) was prepared with acetone and diluted 100‐fold with PBS. A 4% paraformaldehyde‐PBS solution was added, and the sample was allowed to stand for 24 h before washing with PBS and pure water. Mounting agent was added, and a glass coverslip was placed on the sample. The sample was observed by fluorescence microscopy with a WIG longpass fluorescence cube (excitation wavelength: 520–550 nm [green], detectable fluorescence wavelength: >580 nm [red]).

### 2.3 Analysis of fatty acids using liquid chromatography‐mass spectrometry

Comparisons were made between human keratinocytes cultured for 3 days with or without 3.84 mmol/L MVL. Cultured cells were collected, and lipids were extracted using a mixture of solvents (2:1 chloroform:methanol). The lipids were separated by TLC using sequential development with hexane, benzene, and 5:1 chloroform:methanol. This step was followed by development with 80:20:1 hexane:diethyl ether:acetic acid solution, as previously described.^35^ A copper sulfate phosphoric acid solution (copper sulfate [CuSO_4_] was dissolved in 15% phosphoric acid [H_3_PO_4_] solution to produce a 3% [weight/volume] solution) was nebulized and heated, and lipids were stained and imaged. A spot corresponding to the Rf value of the free fatty acids on the TLC plate was scraped off with a knife and transferred to a microtube. Then, 1 ml of mixed solvent was added (2:1 chloroform:methanol), and the mixture was agitated. The supernatant was collected, the solvents were evaporated, and the residue was dissolved in acetonitrile (ACN) for analysis by liquid chromatography‐mass spectrometry (LC–MS).

The parameters for the LC–MS analysis were as follows: LC‐TOF, Waters; LC, Waters Alliance (2695 separation module); TOF, Waters LCT Premier; column, CERI L‐column2 ODS (3.0φ×150 mm, 3 µm, 12 nm); column temperature, 40°C; flow rate, 0.2 ml/min; injection volume, 5 µl; solvent A, 0.1% acetic acid in ACN; A%, 50 (0 min)‐50 (10 min)‐80 (15 min)‐80 (30 min); B, 0.1% acetic acid aqueous solution; C, 0.1% acetic acid in acetone; and gradient conditions (A/B/C), 0 min (85/15/0)‐20 min (85/15/0)‐20.05 min (5/0/95)‐30 min (5/0/95)‐30.05 min (85/15/0) for a total run time of 45 min.

### 2.4 Effects of MVL on protein expression and lipid uptake by keratinocytes cultured with siRNA against ABCA12

Human keratinocytes were seeded onto 35‐mm tissue culture dishes at 500 000 cells/ml. Six microliters of siRNA against ABCA12 (60 pmol; Sigma–Aldrich Corp., St. Louis, MO) + 74 µl of Xfect Reaction Buffer (Tube 1, 80 µl) and 8 µl of Xfect siRNA Transfection Polymer + 72 µl of Xfect Reaction Buffer (Tube 2, 80 µl) were prepared (Clontech Laboratories Inc., TaKaRa Bio USA, Inc., Mountain View, CA). The contents of tube 1 and tube 2 were mixed and then allowed to stand at room temperature for 20 min. The mixed samples were added to 35‐mm tissue culture dishes (160 µl each) and placed in an incubator at 37°C for 24 h. Following culture, a 10% FBS/DMEM control sample and 3.84 mmol/L MVL sample diluted with 10% FBS/DMEM as the MVL sample were prepared. Then, 500 µl of each sample was added to a chamber slide and cultured for 3 days. Nile red stain (prepared at 500 µg/ml with acetone and diluted 100‐fold with PBS) was applied. The sample was washed with PBS and placed in pure water. Following dehydration, each sample was mounted and imaged using a fluorescence microscope with a 20× objective lens (Olympus BX51, Tokyo, Japan).

### 2.5 Measurement of Nile red‐stained granules in cultured human keratinocytes

Human keratinocytes were seeded in 10‐cm dishes and cultured with MVL for 3 days; control cells were also cultured for 3 days. The cells were collected and washed with PBS, homogenized with 0.25 M sucrose solution, and overlaid on a sucrose gradient (1.6 mol/L, 1.4 mol/L, 1.2 mol/L, 1.0 mol/L, 0.8 mol/L, 0.6 mol/L, and 0.4 mol/L). Ultracentrifugation was performed at 28,000 rpm and 4°C for 20 h to recover the sucrose layer. Nile red stain was applied, and a Tali Image‐Based Cytometer (Thermo Fisher Scientific Inc.) was used to measure the number, size, and florescence intensity of the stained granules.

### 2.6 TEWL measurement using the 3D human epidermis model

The 3D human epidermis model (LabCyte EPI‐MODEL) was purchased from Japan Tissue Engineering Co. Ltd. (Aichi, Japan). The aluminum‐wrapped 3D human epidermis model in a cup was fixed to an agar plate, transported at room temperature, transferred to Falcon 24‐well tissue culture plates containing 0.5 ml of dedicated medium, and used for experiments starting the next day.

LabCyte EPI‐MODEL is a 3D human epidermis model in which normal human epidermal cells are cultured in layers and can be used to evaluate the toxicity and irritation effect of test substances. Because the cells retain metabolic activity, the model can be used for dermatological research, such as for measuring production of inflammatory factors and cell growth factors, and for pharmacological assays of various drugs.^36^, ^37^, ^38^ An agarose plate (prepared at the time of shipment of the 3D human epidermis model) was placed in an incubator at 32°C in a room at a temperature of 22°C and humidity of 40%. The human epidermis models were incubated for 5 days with or without 3.84 mmol/L MVL, and these samples were placed in agarose plates. A Tewameter TM210 (Courage and Khazaka Electronic GmbH, Köln, Germany) was used with a 24‐well culture insert probe to measure TEWL, as previously described.^37^

### 2.7 Anti‐ABCA12 antibody and Nile red staining using the 3D human epidermis model

The human epidermis model was cultured for 5 days, after which tissue staining was performed. Tissue‐Tek O.C.T. compound was added to a Tissue‐Tek Cryomold, and the frozen 3D human epidermis model was sectioned to a thickness of 10 µm using a cryostat. After the samples were dried, 4% paraformaldehyde‐PBS was used for fixation. Then, 10% normal goat serum was added, and the sample was incubated for 30 min at room temperature. PBS was used for dilution. The blocking solution was discarded, followed by washing with PBS. The samples were incubated overnight with anti‐ABCA12 polyclonal antibody (Alexa Fluor 488 conjugated, 1 µg/ml). The primary antibody was discarded, followed by a PBS wash. Next, 200 µl of Nile red (adjusted to 500 µg/ml in acetone and further diluted 100‐fold in PBS) stain and DAPI blue fluorescent counterstain diluted 1000‐fold were added, and the mixture was allowed to stand for 1 min at room temperature for nuclear staining. Washing with PBS and then pure water was performed. Following dehydration, the sample was mounted and imaged using a fluorescence microscope with a 20x objective lens.

### 2.8 Analysis of mRNA expression of PPAR β/δ, acetyl‐CoA carboxylase, fatty acid synthase, and ABCA12 in cultured keratinocytes

Keratinocytes were cultured for 1 day in 10% FBS containing 1 ml of DMEM. Then, 10 µl of 5% MVL was added, and the cells were cultured for 3 days. RNA was extracted using an RNA extraction kit (RNeasy Mini kit, Qiagen GmbH, Hilden, Germany). mRNA expression of PPAR β/δ, acetyl‐CoA carboxylase (ACC), fatty acid synthase (FAS), and ABCA12 was measured using real‐time PCR (Applied Biosystems 7900HT, Thermo Fisher Scientific Inc.) with a One Step SYBR PrimeScript RT–PCR Kit II (TaKaRa Bio Inc., Shiga, Japan). GAPDH was chosen as the housekeeping gene. Primers were purchased from Qiagen GmbH. The experiments were conducted in triplicate. For analysis, the target threshold cycle (Ct) was determined by using the difference in Ct value between the housekeeping gene and genes of interest (ΔCt: [Target gene Ct] ‐ [Housekeeping gene Ct]). mRNA expression was compared between genes by calculating differences in ΔCt (ΔΔCt).

### 2.9 Effect of topical application of milky lotion containing MVL on dry skin in humans

We conducted this research following the ethical principles and ethical guidelines for medical research involving human subjects in accordance with the spirit of the Declaration of Helsinki (revised October 2013). After receiving approval from the ethics committee, we sufficiently explained the aim, details, and methods of the study to the 66 female subjects between the ages of 30 and 60 in the informed consent room and then obtained their written consent to participate in the study. The study was conducted on healthy subjects with a low stratum corneum water content, high water evaporation, and low lipid content. Nonhealthy subjects with skin diseases such as atopic dermatitis, psoriasis, ichthyosis, and psoriasis were excluded. The Oriental Beauty Valley Research Institute conducted subject recruitment and management and prepared the test implementation system. Informed consent was obtained from all subjects involved in the study. The TEWL, water content in the stratum corneum, and lipid content of the skin surface were measured in a room with a constant temperature and humidity, and the subjects completed questionnaires regarding the condition of their skin. A randomized, double‐blind, placebo‐controlled, parallel‐group comparison study of topical application of milky lotions was conducted over 2 weeks between January and February 2020. The subjects were randomly allocated into one of two groups based on age, TEWL, water content in the stratum corneum, and lipid content of the skin surface measured before topical application as factors for stratified block randomization, with 33 subjects in each group. We then confirmed no significant differences between the two groups. Both of the test products (active group: a milky lotion containing 0.1% MVL; placebo group: a milky lotion that did not contain active components) were prepared to be indistinguishable in external appearance. The plant extract in the basic formula was 0.1% *Tremella fuciformis* extract. Because the milky lotion with MVL added to the basic formula was used for the active group and the milky lotion with the basic formula was used for the placebo group, the effect of the plant extract showed no difference between the active group and the placebo group. The milky lotion was applied by all subjects to their faces at the amount normally used, twice daily, at morning and night, for 2 weeks. The subjects were not allowed to use any other milky lotion during the study period. The subjects were asked to use their usual cleanser, make‐up remover, lotion, and foundation and were not allowed to switch to other products during the study. After the subjects washed their faces in the face washing room, they acclimated for 30 min in the waiting room. Then, the TEWL, water content in the stratum corneum, and lipid content of the skin surface were measured in the measurement room to evaluate efficacy. The temperature in the face washing room, waiting room, and measurement room was set to 20 ± 1°C, and the humidity was set to 50% ± 2%.

TEWL was measured on the left cheek using a Tewameter TM300 measurement device (Courage + Khazaka Electronic GmbH, Köln, Deutschland). Measurements were made continuously for 60 s, and the mean value of the minimum standard deviation of the reading from the medial 30 s was used. Measurements were conducted three times in the same location, and the mean was recorded. The water content in the stratum corneum was measured on the left cheek using a Corneometer CM 825 stratum corneum water content measurement device (Courage + Khazaka Electronic GmbH). Measurements were conducted six times in the same location, and the mean was recorded. The lipid content on the skin surface was measured on the left cheek using a Sebumeter skin surface lipid content measurement device (Courage + Khazaka Electronic GmbH). Measurements were conducted three times in the same location, and the mean was recorded.

### 2.10 Statistical analysis

**FIGURE 2:**
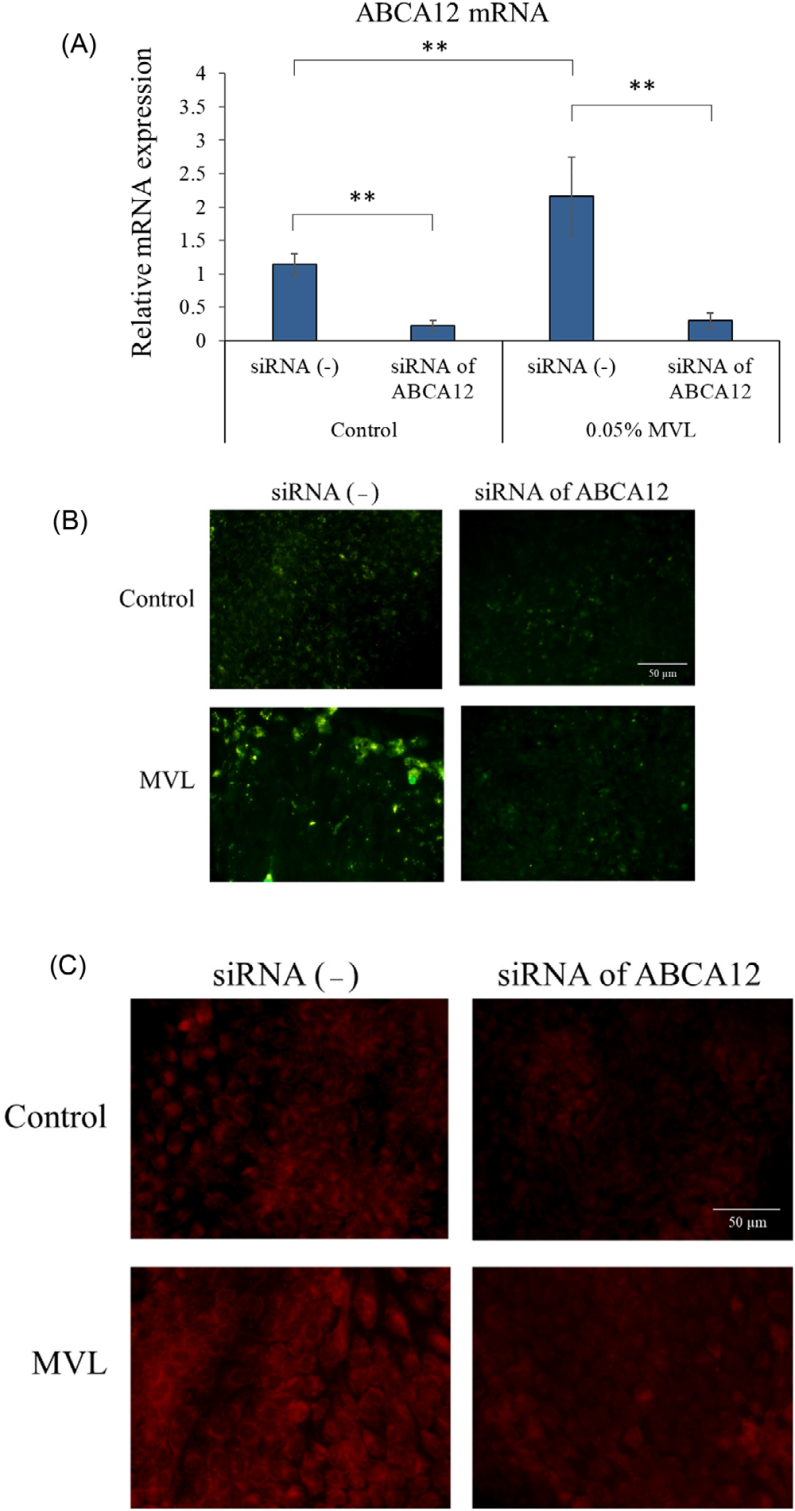
Effects of mevalonolactone (MVL) (3.84 mmol/L) on ABCA12 mRNA expression (A), anti‐ABCA12 immunostaining (B), and Nile red‐positive lipids (C) in human keratinocytes. *n* = 3, mean ± SD. ^∗^ *p* < 0.05; ^∗∗^ *p* < 0.01

**FIGURE 3:**
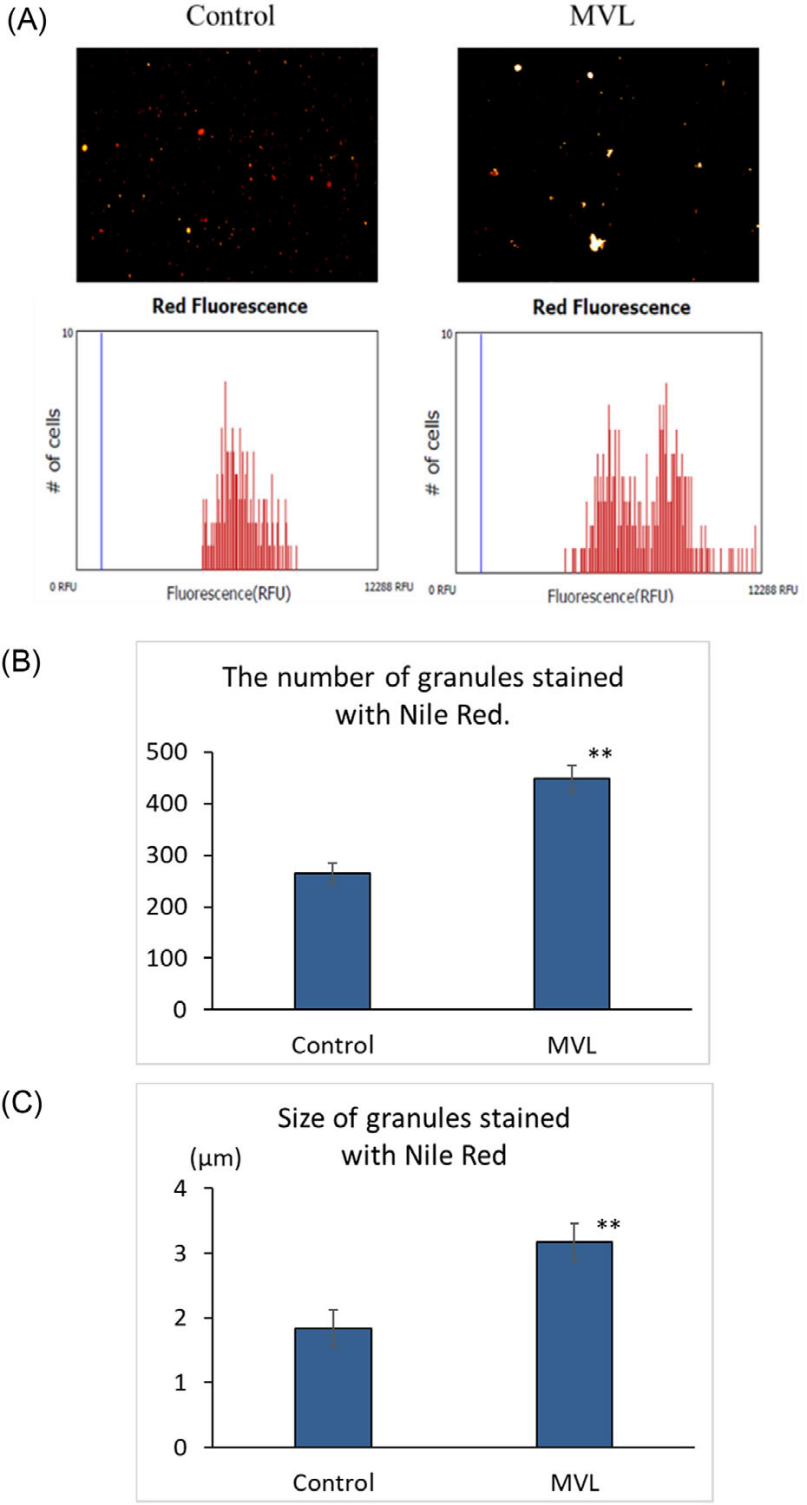
Effects of mevalonolactone (MVL) (3.84 mmol/L) on the fluorescence intensity and (A), number (B), and size (C) of Nile red‐stained granules in cultured human keratinocytes. *n* = 3, mean ± SD. ^∗∗^ *p* < 0.01 versus control

**FIGURE 4:**
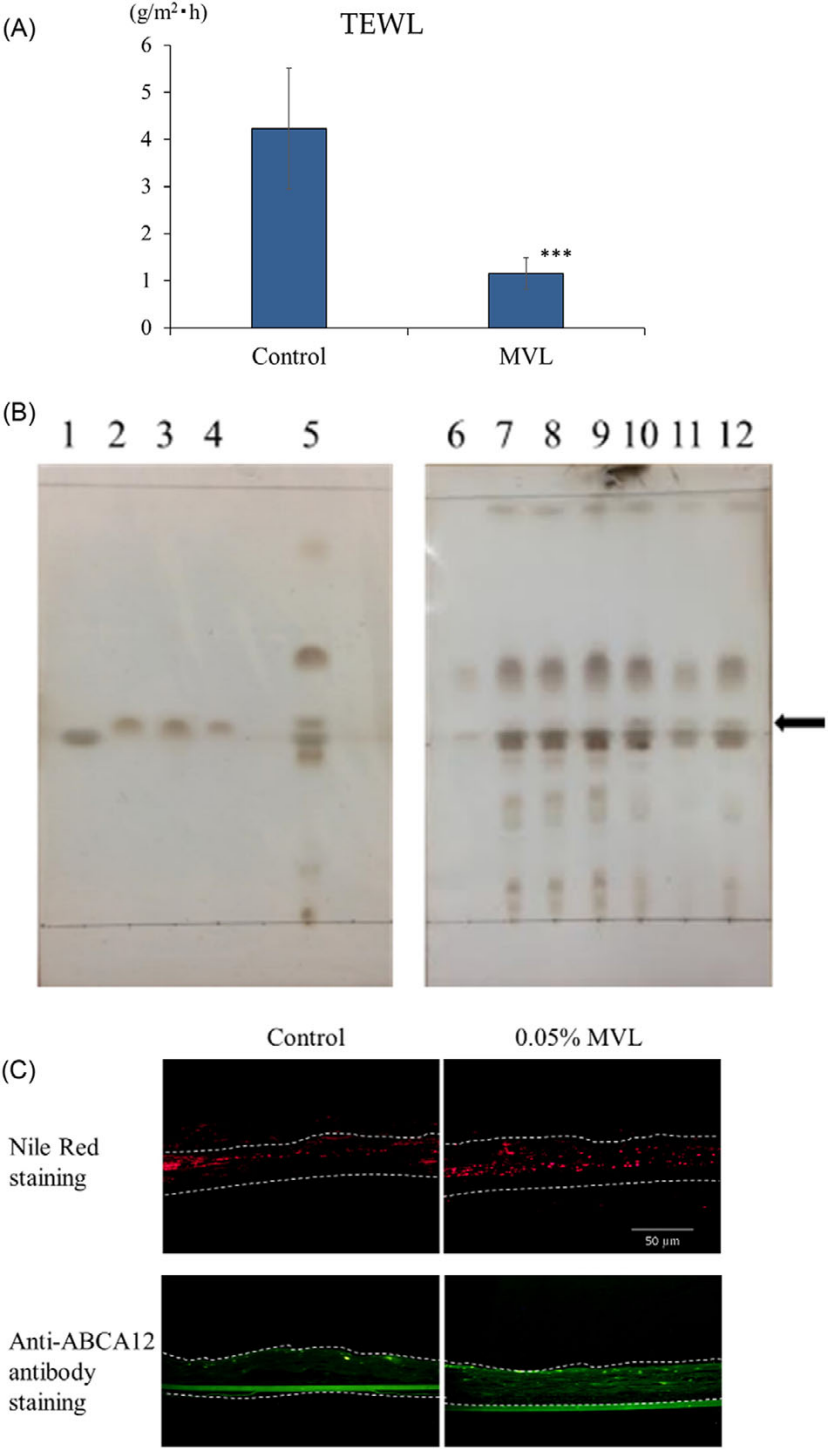
Effects of mevalonolactone (MVL) on transepidermal water loss (TEWL) and on extracted lipids in the 3D human epidermis. (A) Effects of MVL on TEWL in the 3D human epidermis model; *n* = 6, mean ± SD. ^∗∗∗^ *p* < 0.001 versus control. (B) Effects of MVL on extracted lipids from the cultured 3D human epidermis model. The 2:1 chloroform:methanol extract was analyzed using TLC; 1: cholesterol; 2: linoleic acid; 3: linolenic acid; 4: oleic acid; 5: lipid extract from 3D human epidermis model cultured for 5 days with 3.84 mmol/L MVL; 6: triolein; 7–9: lipid extract from 3D human epidermis model cultured for 5 days without MVL (control); 10–12: lipid extract from 3D human epidermis model cultured for 5 days with 3.84 mmol/L MVL. ← indicates free fatty acids. (C) Effects of MVL on Nile red staining and ABCA12 tissue staining in the cultured 3D epidermis model

**FIGURE 5:**
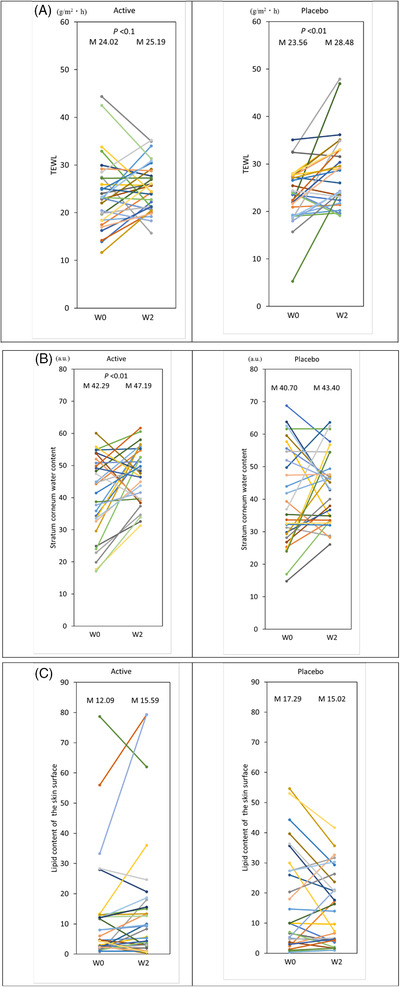
**(A)** Effect of topical application of milky lotion containing mevalonolactone (MVL) on dry skin in humans. Changes in the transepidermal water loss (TEWL) of the skin surface (W0, W2) of the active milky lotion group and the control milky lotion group are displayed as scatter plots; *n* = 30 (active) and *n* = 29 (placebo), *p < 0.05 between W0 and W2 in the placebo group. M represents the mean value. **(B)** Effect of topical application of milky lotion containing MVL on dry skin in humans. Changes in the stratum corneum water content of the skin (W0, W2) of the active milky lotion group and the control milky lotion group are displayed as scatter plots; *n* = 30 (active) and *n* = 29 (placebo), *p < 0.01 between W0 and W2 in the active group. M represents the mean value. **(C)** Effect of topical application of milky lotion containing MVL on dry skin in humans. Changes in lipid content on the skin surface (W0, W2) of the active milky lotion group and the control milky lotion group are displayed as scatter plots; *n* = 30 (active) and *n* = 29 (placebo). M represents the mean value.

The data shown in Figure 2 were analyzed using two‐way analysis of variance and BellCurve Excel Statistics (Social Survey Research Information Co., Ltd., Tokyo, Japan). Tukey's test was employed for multiple comparisons. For the data presented in Figures 3, 4, and 5, two‐tailed t tests were performed. A *p* value of <0.05 was considered statistically significant.

Regarding efficacy evaluation items in human trials, summary statistics of the results of measurements were calculated, and statistical analysis was conducted using the following methods. The significance level for tests was taken to be 5%, and the tendency for improvement for tests was taken to be greater than 5% and up to 10%. The data were statistically analyzed using two‐tailed paired *t* tests comparing the same subject with respect to measured values before and after topical application. The data were statistically analyzed using two‐tailed unpaired *t* tests comparing the group in which the milky lotion that was applied contained 0.1% MVL (the active group) and the group in which the placebo milky lotion was applied (the placebo group).

## 3 RESULTS

### 3.1 Effects of MVL on ABCA12 mRNA expression and Nile red‐positive lipid content in keratinocytes

ABCA12 mRNA levels increased in keratinocytes cultured for 3 days with 3.84 mmol/L MVL (Figure 2A). When siRNA against ABCA12 was added to the sample, ABCA12 mRNA expression decreased (Figure 2A). Fluorescence microscopy of samples stained with anti‐human ABCA12 antibodies revealed stronger fluorescence in the 3.84 mmol/L MVL sample than in the control (Figure 2B). In both the control and MVL‐treated samples, addition of siRNA targeting ABCA12 decreased anti‐ABCA12 immunofluorescence (Figure 2B). This observation confirmed that addition of siRNA against ABCA12 inhibits ABCA12 protein levels. Lipid staining with Nile red is shown in Figure 2C. Compared with the control, the MVL‐treated sample showed stronger fluorescence (Figure 2C). Among the controls, samples with and without siRNA targeting ABCA12 were compared, and stained granules were present in samples without siRNA, whereas samples with siRNA showed no granular inclusions. Thus, the inhibition of ABCA12 causes disappearance of granularly stained regions of these cells.

### 3.2 Effects of MVL on the free fatty acid content of cultured keratinocytes

A sucrose density gradient protocol was used to fractionate organelles from cultured keratinocytes, which were then stained with Nile red, and the Nile red‐stained granules were quantified with a Tali Image‐Based Cytometer. The results showed that cells cultured with 3.84 mmol/L MVL had larger and more strongly fluorescent Nile red‐stained granules than control cells (Figure 3).

### 3.3 Analysis of fatty acids using LC–MS

Keratinocytes were cultured for 3 days with 3.84 mmol/L MVL and treated with chloroform/methanol to obtain an extract. TLC was used to resolve the extract, and LC–MS was applied to assess the TLC spot corresponding to unsaturated fatty acids. MS analysis of the spot revealed the saturated acids to be primarily composed of C14:0 (myristic acid), C16:0 (palmitic acid), and C18:0 (stearic acid); unsaturated fatty acids were primarily consisted of C16:1 (palmitoleic acid), C18:1 (oleic acid), and C18:2 (linoleic acid). Comparison of the lipid content of chloroform/methanol extracts for cells cultured with MVL versus cells without MVL (control) is shown in Table 1. Addition of MVL to the cell culture increased levels of C14:0, C16:0, C16:1, C18:0, and C18:1. For C14:0, C16:0, and C16:1, levels were more than three times higher in cells cultured with MVL than in control cells.

**TABLE 1:** Comparison of the fatty acid content in chloroform:methanol extracts from cells cultured with mevalonolactone (MVL) versus without MVL (control)

	Control	MV 3.84 mmol/L	Fold
C12:0	0.04	0.06	1.53
C14:0	0.17	0.79	4.60
C16:0	1.88	6.78	3.60
C16:1	1.81	6.00	3.31
C18:0	1.13	2.38	2.10
C18:1	5.29	10.11	1.91
C18:2	0.10	0.13	1.28
C13:0	1.00	1.00	1.00

### 3.4 Effects of MVL on TEWL and the free fatty acid content in the 3D human epidermis model

The 3D human epidermis model cultured for 5 days with 3.84 mmol/L MVL showed significantly decreased stratum corneum water loss, with a TEWL of 27% compared with that of the control value (Figure 4A). The free fatty acid content of the 3D human epidermis model cultured for 5 days with 3.84 mmol/L MVL was significantly greater than that of control (Figure 4B). Increased numbers of Nile red‐stained granules were observed in the 3D human epidermis model cultured with MVL compared with the control (Figure 4C). Furthermore, anti‐ABCA12 antibody fluorescence was increased in the epidermis model cultured with MVL compared with the control (Figure 4C).

### 3.5 Effect of topical application of milky lotion containing MVL on dry skin in humans

**FIGURE 6:**
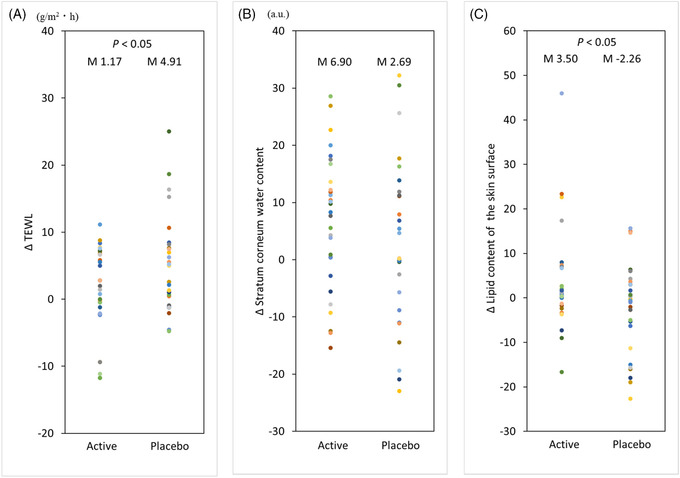
Effect of topical application of milky lotion containing mevalonolactone (MVL) on dry skin in humans. Changes in the transepidermal water loss (TEWL) (A), water content in the stratum corneum (B), and lipid content of the skin surface (C) were analyzed; the results are shown as scatter plots. Significant difference tests were performed between the active and control milky lotion groups; *n* = 30 (active) and *n* = 29 (placebo). *p < 0.05 between active and placebo in the ΔTEWL and Δ lipid content of the skin surface. M represents the mean value.

In the active group, two subjects dropped out of the study, and one was excluded from the analysis because the lipid content was 0; four subjects in the placebo group dropped out. The results for 30 subjects in the active group and 29 subjects in the placebo group were used for statistical analysis. The age of the active group was 45.3 ± 6.5 (mean ± SD), and that of the placebo group was 45.9 ± 7.6 (mean ± SD). There were no significant differences between the active and placebo groups before topical application in terms of age, TEWL, water content in the stratum corneum, or lipid content of the skin surface. The TEWL results are shown in Figure 5A, increasing significantly in the placebo group at week 2 (W2) compared to the start (W0); in the active group, W2 tended to be lower than W0. Comparison between the change in TEWL before and after application showed a significant improvement in TEWL in the active group compared to the placebo group (Figure 6A).

**FIGURE 7:**
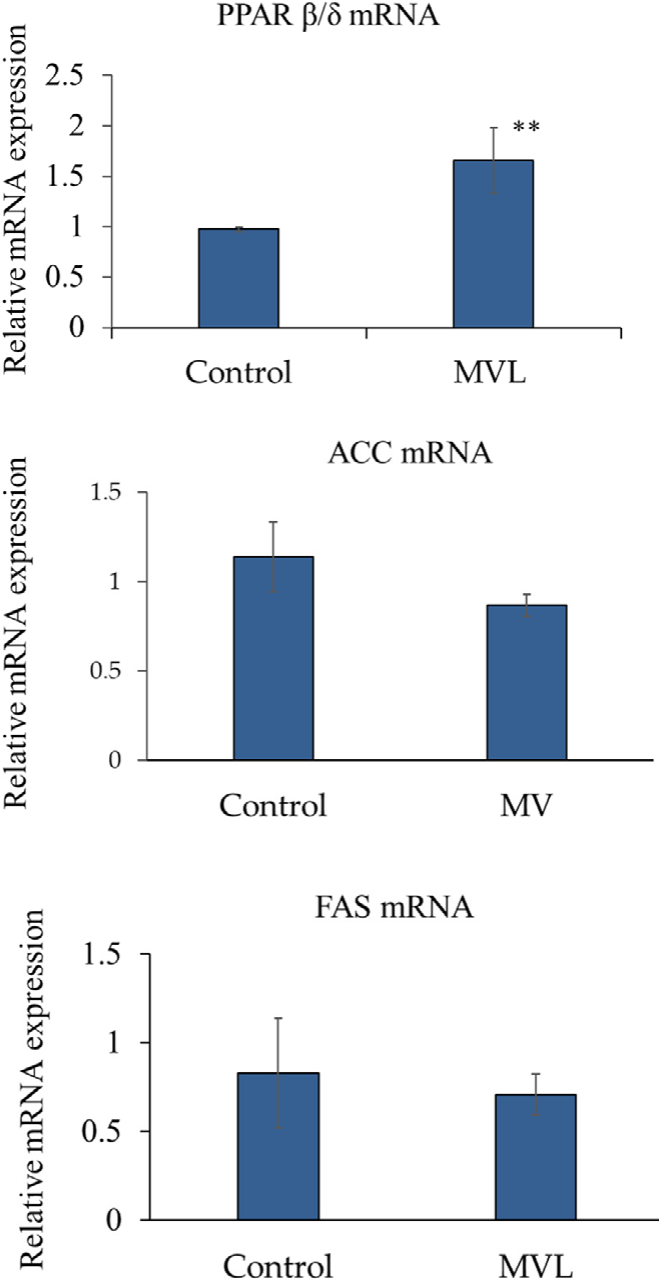
Effects of mevalonolactone (MVL) (3.84 mmol/L) on mRNA expression of peroxisome proliferator‐activated receptor β/δ (PPAR β/δ), acetyl‐CoA carboxylase (ACC), and fatty acid synthase (FAS) in cultured keratinocytes. *n* = 3, mean ± SD. **p < 0.01 versus the control; ^+^ *p* < 0.1 versus the control.

The water content in the stratum corneum increased significantly in the active group at week 2 (W2) compared to the beginning (W0), but there was no change in the placebo group (Figure 5B). Comparison between the change in stratum corneum water content before and after application showed no significant difference between the groups (Figure 6B).

The lipid content of the skin surface was unchanged in the second week (W2) compared to the start (W0) in both groups (Figure 5C). In comparison of the changes in lipid content of the skin surface before and after application, the active group showed a significant increase in lipid content compared to the placebo group (Figure 6C).

The above results of this randomized, double‐blind, placebo‐controlled, parallel‐group comparison study in humans indicate that a milky lotion containing 0.1% MVL is able to alleviate the deterioration of the barrier function of the stratum corneum that occurs during winter. It should be noted that in the human test, the concentration was doubled because the stratum corneum inhibits MVL permeability.

### 3.6 Analysis of mRNA expression of PPAR β/δ, ACC and FAS in cultured keratinocytes

In human keratinocytes cultured for 3 days, mRNA expression levels of PPAR β/δ increased 1.7‐fold with addition of 3.84 mmol/L MVL compared to the control, representing a statistically significant increase (Figure 7). There were no differences in ACC or FAS mRNA expression with or without 3.84 mmol/L MVL application (Figure 7).

## 4 DISCUSSION

Quantification of Nile red‐stained granules showed that keratinocytes cultured with 3.84 mmol/L MVL displayed more, larger, and more strongly fluorescent Nile red‐stained granules than the control cells. The Nile red‐stained granules in the present study were thought to be lamellar granule complexes, as the size of the lamellar body has been reported to be 200–300 nm,^39^ suggesting that MVL increases transport of fatty acids to lamellar granules.

Analysis of free fatty acids with LC–MS revealed the saturated fatty acids to be composed of myristic acid, palmitic acid, and stearic acid and the unsaturated fatty acids to be composed of palmitoleic acid (C16:1), oleic acid (C18:1) and linoleic acid (C18:2). Addition of MVL to the cell culture increased levels of myristic acid (C14:0), palmitic acid (C16:0), palmitoleic acid (C16:1), stearic acid (C18:0), and oleic acid (C18:1). MVL increased levels of not only saturated fatty acids but also monounsaturated fatty acids. Free fatty acids that could be separated from the human stratum corneum include lignoceric acid (C24:0), hexacosanoic acid (C26:0), and stearic acid (C18:0), as well as palmitic acid (C16:0), arachidic acid (C20:0), and behenic acid (C22:0) in small amounts. Oleic acid (C18:1) and linoleic acid (C18:2) are examples of unsaturated fatty acids found in the stratum corneum.^40^ These fatty acids are thought to be stored within lamellar granules. Stratum granulosum cells contain many lamellar granules,^7^ and in the present study, MVL appeared to increase both the number and size of lamellar granules. Free fatty acids stored in lamellar granules are, along with ceramide, secreted between keratinocytes and thus become intercellular lipids. Therefore, MVL can prevent deterioration of stratum corneum barrier function.

PPAR β/δ are also expressed in the epidermis, and loss of PPAR β/δ is reported to lead to inflammation or decreased barrier function.^41^, ^42^ In examining the effects of MVL on PPAR β/δ mRNA expression in cultured keratinocytes, we found that addition of MVL increased expression. As long‐chain fatty acids are ligands for PPAR β/δ, we infer that MVL enhances PPAR β/δ levels by increasing the free fatty acid content in keratinocytes. Along with the increase in PPAR β/δ expression observed in keratinocytes cultured with MVL, addition of MVL caused an increase in the number, size, and fluorescence of lamellar granules (which include unsaturated fatty acids) when cells were fractionated with sucrose. MVL did not appear to promote synthesis of fatty acids. On the other hand, PPAR β/δ promotes uptake of fatty acids,^43^ suggesting that the increase in intracellular fatty acids by MVL involves an increase in PPAR β/δ. Because MVL increased the free fatty acid content and lamellar granule formation in cultured keratinocytes, it can be inferred that MVL promotes free fatty acid uptake in keratinocytes, increasing expression of PPAR β/δ and ultimately leading to lamellar granule formation. Based on the results from the 3D human epidermis model and human testing, which demonstrate that MVL decreases evaporative water loss from the stratum corneum, this molecule may improve epidermal barrier function.

It has been reported that cholesterol/lovastatin is associated with hyperkeratosis in sweat pore keratosis.^34^ Statins decrease the number and internal contents of epidermal lamellar bodies and disrupt the lamellar bilayer structure between cells.^44^, ^45^ Statins, which inhibit the action of HMG‐CoA reductase, inhibit conversion of HMG‐CoA to MVA and decrease MVA and intermediates in the MVA pathway, thereby reducing blood levels of the end product, cholesterol. Because ACC and FAS, which are necessary for fatty acid synthesis, increase in conjunction with barrier disruption,^46^ the increase in the number and internal contents of keratinocyte lamellar bodies caused by MVL is unlikely to involve intermediate metabolites of the MVA pathway or the cholesterol end product.

The water content in the stratum corneum was increased in the MVL application group after application for 2 weeks compared with the placebo group, but no significant difference was observed between the groups after 2 weeks. We propose that this result is due to the use of the Corneometer, which measures the water content up to the depth of the stratum corneum, which is rich in water. In the future, it will be necessary to use the Skicon‐200EX instrument, which measures the water content on the skin surface and the shallow part of the stratum corneum.^47^ Furthermore, an increase in the lipid content was confirmed in the MVL application group, but it is believed that not only the lipids derived from the sebaceous glands but also those derived from the stratum corneum were measured. The TEWL of the placebo group increased significantly at 2 weeks, and the barrier function of the stratum corneum worsened, but the TEWL of the active group did not change at 2 weeks. The increase in TEWL in the placebo group may have occurred because the test was conducted in winter, when the temperature and humidity are lower. The TEWL of the active group did not change after 2 weeks, which was thought to be because MVL enhanced barrier function. On the other hand, the TEWL of the active group was lower than that of the placebo group after 2 weeks, and there was a significant difference between the two groups, suggesting that MVL is effective in preventing barrier function deterioration.

Spontaneous mutations in or other damage to the ABCA12 gene interfere with intercellular lipid transport and proper lipid storage within lamellar granules.^25^ Eventually, intercellular lipid stores are diminished, leading to impaired barrier function. Therefore, ABCA12 is critical for maintaining the intercellular lipid content and thus proper barrier function in the stratum corneum. Cultured keratinocytes with and without siRNA against ABCA12 were compared. Samples without added siRNA displayed stained granules, whereas samples with siRNA did not. Thus, we conclude that inhibition of ABCA12 decreases granular staining. Because ABCA12 has been reported to be involved in transport of lipids,^26^ we suggest that repression of the ABCA12 gene inhibits normal lipid transport to lamellar granules, thus resulting in loss of the granular staining region. Furthermore, we reasoned that inhibition of ABCA12 led to impaired lipid transport into the lamellar granules, causing a lack of granular staining. The ABCA12 level reportedly increases in response to PPAR α and PPAR β/δ.^27^ We can thus infer that in keratinocytes cultured with MVL, the PPAR β/δ level was increased, leading to increased expression of ABCA12 mRNA and protein and ultimately resulting in an increased lipid content in lamellar granules. Future analysis of ABCA12 and PPAR in dry skin should be conducted in clinical trials.

## 5 CONCLUSIONS

The data obtained with cultured cells suggest that MVL can alleviate deterioration of stratum corneum barrier function by increasing ABCA12 through an increase in the PPAR β/δ level, facilitation of fatty acid transport to lamellar granules, and a higher fatty acid content in the stratum granulosum, which ultimately increases the level of stratum corneum intercellular lipids. Thus, it is suggested that milky lotions containing MVL promote the epidermal barrier function of human dry skin.

## CONFLICT OF INTEREST

The authors declare that there is no conflict of interest that could be perceived as prejudicing the impartiality of the research reported.

## ETHICAL APPROVAL STATEMENT

The authors conducted this research following ethical principles and ethical guidelines for medical research involving human subjects in accordance with the spirit of the Declaration of Helsinki (revised October 2013). After receiving approval from the ethics committee, they sufficiently explained the aim, details, and methods of the study to the 66 female subjects between the ages of 30 and 60 in the informed consent room and then obtained their written consent to participate in the study. The study was conducted using healthy subjects with a low stratum corneum water content, high water evaporation, and low lipid content. Nonhealthy subjects with skin diseases such as atopic dermatitis, psoriasis, ichthyosis, and psoriasis were not included in the study. The Oriental Beauty Valley Research Institute conducted the subject recruitment and management and prepared the test implementation system. Informed consent was obtained from all subjects involved in the study.

## Data Availability

The data that support the findings of this study are available from the corresponding author upon reasonable request.

## References

[1] Norlén L , Nicander I , Lundh Rozell B , Ollmar S , Forslind B . Inter‐ and intra‐individual differences in human stratum corneum lipid content related to physical parameters of skin barrier function in vivo. J Invest Dermatol. 1999;112(1):72‐77. 9886267 10.1046/j.1523-1747.1999.00481.x

[2] Wertz P , Norlén L . “Confidence intervals” for the “true” lipid composition of the human skin barrier? In: Forslind B , Lindberg M, eds. Skin, Hair and Nails, Structure and Function. Marcel Dekker Inc; 2003:85‐106.

[3] Feingold KR , Elias PM . Role of lipids in the formation and maintenance of the cutaneous permeability barrier. Biochim Biophys Acta. 2014;1841(3):280‐294. 24262790 10.1016/j.bbalip.2013.11.007

[4] Groen D , Poole DS , Gooris GS , Bouwstra JA . Investigating the barrier function of skin lipid models with varying compositions. Eur J Pharm Biopharm. 2011;79(2):334‐342. 21658448 10.1016/j.ejpb.2011.05.007

[5] Schmuth M , Man MQ , Weber F , et al. Permeability barrier disorder in Niemann‐Pick disease: sphingomyelin‐ceramide processing required for normal barrier homeostasis. J Invest Dermatol. 2000;115(3):459‐466. 10951284 10.1046/j.1523-1747.2000.00081.x

[6] Odland GF . A submicroscopic granular component in human epidermis. J Invest Dermatol. 1960;34:11‐15 14428292

[7] Wolff K , Holubar K . Odland‐Körper (Membrane Coating Granules, Keratinosomen) als epidermale Lysosomen. Ein elektronenmikroskopisch‐cytochemischer Beitrag zum Verhornungsprozess der Haut [Odland bodies (Membrane coating granules, keratinosomes) as epidermal lysosomes. An electron microscopic‐cytochemical contribution on the cornification process of the skin]. Arch Klin Exp Dermatol. 1967;231(1):1‐19. 5592065

[8] Freinkel RK , Traczyk TN . Lipid composition and acid hydrolase content of lamellar granules of fetal rat epidermis. J Invest Dermatol. 1985;85(4):295‐298. 4045218 10.1111/1523-1747.ep12276831

[9] Lee SH , Elias PM , Proksch E , Menon GK , Mao‐Quiang M , Feingold KR . Calcium and potassium are important regulators of barrier homeostasis in murine epidermis. J Clin Invest. 1992;89(2):530‐538. 1737844 10.1172/JCI115617 PMC442884

[10] Wertz PW , Madison KC , Downing DT . Covalently bound lipids of human stratum corneum. J Invest Dermatol. 1989;92(1):109‐111. 2909622 10.1111/1523-1747.ep13071317

[11] Robson KJ , Stewart ME , Michelsen S , Lazo ND , Downing DT . 6‐Hydroxy‐4‐sphingenine in human epidermal ceramides. J Lipid Res. 1994;35(11):2060‐2068. 7868984

[12] Uchiyama N , Yamamoto A , Kameda K , Yamaguchi H , Ito M . The activity of fatty acid synthase of epidermal keratinocytes is regulated in the lower stratum spinousum and the stratum basale by local inflammation rather than by circulating hormones. J Dermatol Sci. 2000;24(2):134‐141 11064249 10.1016/s0923-1811(00)00088-8

[13] Mao‐Qiang M , Elias PM , Feingold KR . Fatty acids are required for epidermal permeability barrier function. J Clin Invest. 1993;92(2):791‐798. 8102380 10.1172/JCI116652 PMC294916

[14] Ziboh VA , Miller CC . Essential fatty acids and polyunsaturated fatty acids: significance in cutaneous biology. Annu Rev Nutr. 1990;10:433‐450. 2200473 10.1146/annurev.nu.10.070190.002245

[15] Burr GO , Burr MM . On the nature and role of fatty acids essential in nutrition. J Biol Chem. 1930;86:587‐621.

[16] Khnykin D , Miner JH , Jahnsen F . Role of fatty acid transporters in epidermis: implications for health and disease. Dermatoendocrinol. 2011;3(2):53‐61. 21695012 10.4161/derm.3.2.14816 PMC3117002

[17] Skolnik P , Eaglstein WH , Ziboh VA . Human essential fatty acid deficiency: treatment by topical application of linoleic acid. Arch Dermatol. 1977;113(7):939‐941. 406855

[18] Campbell KL . Fatty acid supplementation and skin disease. Vet Clin North Am Small Anim Pract. 1990;20(6):1475‐1486. 2251737 10.1016/s0195-5616(90)50156-4

[19] Ziboh VA , Miller CC , Cho Y . Metabolism of polyunsaturated fatty acids by skin epidermal enzymes: generation of antiinflammatory and antiproliferative metabolites. Am J Clin Nutr. 2000;71(1 Suppl):361S‐366S. 10617998 10.1093/ajcn/71.1.361s

[20] Horrobin DF . Essential fatty acid metabolism and its modification in atopic eczema. Am J Clin Nutr. 2000;71(1 Suppl):367S‐372S. 10617999 10.1093/ajcn/71.1.367s

[21] McCusker MM , Grant‐Kels JM . Healing fats of the skin: the structural and immunologic roles of the omega‐6 and omega‐3 fatty acids. Clin Dermatol. 2010;28(4):440‐451. 20620762 10.1016/j.clindermatol.2010.03.020

[22] Sala‐Vila A , Miles EA , Calder PC . Fatty acid composition abnormalities in atopic disease: evidence explored and role in the disease process examined. Clin Exp Allergy. 2008;38(9):1432‐1450. 18665842 10.1111/j.1365-2222.2008.03072.x

[23] Ziboh VA , Miller CC , Cho Y . Significance of lipoxygenase‐derived monohydroxy fatty acids in cutaneous biology. Prostaglandins Other Lipid Mediat. 2000;63(1‐2):3‐13. 11104337 10.1016/s0090-6980(00)00093-9

[24] Ziboh VA , Cho Y , Mani I , Xi S . Biological significance of essential fatty acids/prostanoids/lipoxygenase‐derived monohydroxy fatty acids in the skin. Arch Pharm Res. 2002;25(6):747‐758. 12510822 10.1007/BF02976988

[25] Lefévre C , Audebert S , Jobard F , et al. Mutations in the transporter ABCA12 are associated with lamellar ichthyosis type 2. Hum Mol Genet. 2003;12(18):2369‐2378. 12915478 10.1093/hmg/ddg235

[26] Sakai K , Akiyama M , Sugiyama‐Nakagiri Y , McMillan JR , Sawamura D , Shimizu H . Localization of ABCA12 from Golgi apparatus to lamellar granules in human upper epidermal keratinocytes. Exp Dermatol. 2007;16(11):920‐326. 17927575 10.1111/j.1600-0625.2007.00614.x

[27] Jiang YJ , Lu B , Kim P , et al. PPAR and LXR activators regulate ABCA12 expression in human keratinocytes. J Invest Dermatol. 2008;128(1):104‐109. 17611579 10.1038/sj.jid.5700944

[28] Fowler SD , Brown WJ , Warfel J , Greenspan P . Use of nile red for the rapid in situ quantitation of lipids on thin‐layer chromatograms. J Lipid Res. 1987;28(10):1225‐1232. 3681147

[29] Boumelhem BB , Pilgrim C , Zwicker VE , et al. Intracellular flow cytometric lipid analysis ‐ a multiparametric system to assess distinct lipid classes in live cells. J Cell Sci. 2022;135(5):jcs258322. 34114626 10.1242/jcs.258322

[30] Greenspan P , Mayer EP , Fowler SD . Nile red: a selective fluorescent stain for intracellular lipid droplets. J Cell Biol. 1985;100(3):965‐973 3972906 10.1083/jcb.100.3.965 PMC2113505

[31] Amen N , Mathow D , Rabionet M , et al. Differentiation of epidermal keratinocytes is dependent on glucosylceramide:ceramide processing. Hum Mol Genet. 2013;22(20):4164‐4179. 23748427 10.1093/hmg/ddt264

[32] Haratake A , Ikenaga K , Katoh N , Uchiwa H , Hirano S , Yasuno H . Topical mevalonic acid stimulates de novo cholesterol synthesis and epidermal permeability barrier homeostasis in aged mice. J Invest Dermatol. 2000;114(2):247‐252. 10651982 10.1046/j.1523-1747.2000.00875.x

[33] Proksch E , Holleran WM , Menon GK , Elias PM , Feingold KR . Barrier function regulates epidermal lipid and DNA synthesis. Br J Dermatol. 1993;128(5):473‐482. 8504036 10.1111/j.1365-2133.1993.tb00222.x

[34] Atzmony L , Lim YH , Hamilton C , et al. Topical cholesterol/lovastatin for the treatment of porokeratosis: a pathogenesis‐directed therapy. J Am Acad Dermatol. 2020;82(1):123‐131. 31449901 10.1016/j.jaad.2019.08.043 PMC7039698

[35] Maeda K , Yamazaki J , Okita N , Shimotori M , Igarashi K , Sano T . Mechanism of cuticle hole development in human hair due to UV‐radiation exposure. Cosmetics 2008;5(2):24.

[36] Sugiura K , Muro Y , Futamura K , et al. The unfolded protein response is activated in differentiating epidermal keratinocytes. J Invest Dermatol. 2009;129(9):2126‐2135. 19282840 10.1038/jid.2009.51

[37] Morimoto H , Gu L . Zeng L , Maeda K . Amino carbonylation of epidermal basement membrane inhibits epidermal cell function and is suppressed by methylparaben. Cosmetics 2017;4(4):38.

[38] Hashimoto M , Maeda K . New functions of low‐molecular‐weight hyaluronic acid on epidermis filaggrin production and degradation. Cosmetics 2021;8(4):118.

[39] Joshi R . Learning from eponyms: George F. Odland and Odland bodies. Indian Dermatol Online J. 2014;5:334‐338. 25165659 10.4103/2229-5178.137794 PMC4144227

[40] Norlén L , Nicander I , Lundsjö A , Cronholm T , Forslind B . A new HPLC‐based method for the quantitative analysis of inner stratum corneum lipids with special reference to the free fatty acid fraction. Arch Dermatol Res. 1998;290(9):508‐516. 9808345 10.1007/s004030050344

[41] Schmuth M , Haqq CM , Cairns WJ , et al. Peroxisome proliferator‐activated receptor (PPAR)‐beta/delta stimulates differentiation and lipid accumulation in keratinocytes. J Invest Dermatol. 2004;122(4):971‐983. 15102088 10.1111/j.0022-202X.2004.22412.x

[42] Man MQ , Choi EH , Schmuth M , et al. Basis for improved permeability barrier homeostasis induced by PPAR and LXR activators: liposensors stimulate lipid synthesis, lamellar body secretion, and post‐secretory lipid processing. J Invest Dermatol. 2006;126(2):386‐392. 16374473 10.1038/sj.jid.5700046

[43] Holst D , Luquet S , Nogueira V , Kristiansen K , Leverve X , Grimaldi PA . Nutritional regulation and role of peroxisome proliferator‐activated receptor δ in fatty acid catabolism in skeletal muscle. Biochim Biophys Acta. 2003;1633(1):43‐50. 12842194 10.1016/s1388-1981(03)00071-4

[44] Menon GK , Feingold KR , Mao‐Qiang M , Schaude M , Elias PM . Structural basis for the barrier abnormality following inhibition of HMG CoA reductase in murine epidermis. J Invest Dermatol. 1992;98(2):209‐219. 1732385 10.1111/1523-1747.ep12555880

[45] Ito M , Fujiwara H , Maruyama T , Oguro K , Ishihara O , Sato Y . Morphogenesis of the cornoid lamella: histochemical, immunohistochemical, and ultrastructural study of porokeratosis. J Cutan Pathol. 1991;18(4):247‐256. 1939783 10.1111/j.1600-0560.1991.tb01231.x

[46] Harris IR , Farrell AM , Grunfeld C , Holleran WM , Elias PM , Feingold KR . Permeability barrier disruption coordinately regulates mRNA levels for key enzymes of cholesterol, fatty acid, and ceramide synthesis in the epidermis. J Invest Dermatol. 1997;109(6):783‐787. 9406821 10.1111/1523-1747.ep12340962

[47] Tagami H . Electrical measurement of the hydration state of the skin surface in vivo. Br J Dermatol. 2014;171(Suppl 3):29‐33. 10.1111/bjd.13245 25234175

